# The Emerging Role of B Cells and Tertiary Lymphoid Structures in Bladder Cancer

**DOI:** 10.1007/s11934-025-01314-z

**Published:** 2025-12-29

**Authors:** Andrew Penunuri, Eliyahu Greenberg, John Phillips, Andrew B. Katims

**Affiliations:** Department of Urology, New York Medical College, 19 Skyline Drive, Hawthrone, NY USA

**Keywords:** Bladder cancer, B cells, Tertiary lymphoid structures, Tumor microenvironment

## Abstract

**Purpose of Review:**

To provide a comprehensive review of new and emerging literature on B cells and tertiary lymphoid structures (TLS) in urothelial cancer.

**Recent Findings:**

The presence of certain subsets of B cells and TLS in MIBC are associated with improved outcomes and response to immunotherapy. However, the role in NMIBC is less clear. TLS in NMIBC is likely associated with more aggressive disease but may also be associated with improved outcomes to intravesical BCG.

**Summary:**

This review outlines an emerging potential biomarker (TLS) for predictive and prognostic outcomes for patients with urothelial cancer receiving either intravesical BCG or systemic immunotherapy. B cells play a complex role in the tumor immune microenvironment and can be either anti- or pro-tumor. Activated B cells form TLS which lead to a robust response to immunotherapy. This has led some investigators to attempt therapeutic manipulation to induce TLS in otherwise immunologically “cold” tumors.

## Introduction

Immunotherapy (IO) has emerged as a critical tool in the management of urothelial cancer. For decades, intravesical Bacillus Calmette-Guérin (BCG), often considered one of the earliest IOs, has been used in the management of non-muscle invasive bladder cancer (NMIBC). More recently, systemic immune checkpoint inhibitors (ICIs) have provided efficacious responses in some patients with advanced or muscle-invasive disease [1] and may have a role in BCG-unresponsive NMIBC [2]. The application of ICI in first line, BCG-naïve NMIBC is also evolving with the recently published CREST and POTOMAC trials, both showing improved event free survival in patients treated with ICI + BCG compared to BCG alone [3, 4]. However, the clinical benefit of local and systemic IO varies significantly at the patient level, leaving opportunity for a personalized or precision medicine approach to therapeutic choice. Identifying immune correlates between response and resistance within the tumor microenvironment (TME) remains a focus of researchers and clinicians to better optimize treatment choice [5].

In recent years, molecular characterization of bladder tumors has expanded and allowed for large scale transcriptomic profiling which has led to classification groupings. Specifically, muscle-invasive bladder cancer (MIBC) can be divided into 6 molecular subtypes: luminal papillary (24%), luminal non-specified (8%), luminal unstable (15%), stroma-rich (15%), basal/squamous (35%), and neuroendocrine-like (3%). Each subtype differs significantly in oncologic mechanisms and immune infiltrate and correlates with histologic/clinical characteristics, response to therapy, and outcome [6]. While there is a direct clinical correlation to consensus classification, practical implementation has been challenging due to high costs and limited availability of sequencing at many practices. Histological characteristics and immunohistochemistry alone have provided some promise of widespread clinical application of consensus clustering [7] but is yet to be seen in everyday practice.

The TME of bladder cancer is highly heterogeneous, ranging from inflamed “hot” tumors with abundant immune infiltration to “cold” tumors with sparse immune cells [8]. Inflamed tumors, often associated with basal and luminal-infiltrated molecular subtypes, are characterized by high T cell and B cell infiltration, expression of immune checkpoints, and the presence of tertiary lymphoid structures (TLS). In contrast, cold tumors, such as the luminal-papillary subtype, exhibit low immune infiltration and are less responsive to immunotherapy [9]. Beyond the presence of immune cells, the relative abundance of specific immune cells present, spatial relationship within, adjacent to, or excluded from tumors, and receptor specific cell-cell interactions may guide response or resistance to immunotherapy [10, 11].

T cells, particularly CD8^+^ cytotoxic T lymphocytes, are established mediators of antitumor immunity and are central to the efficacy of ICI [12]. However, emerging evidence indicates that B cells and TLS also play critical roles in modulating antitumor responses. B cells within TLS can present antigens, produce antibodies, and support T cell activation, while regulatory B cells (B_regs_) may suppress immunity and promote tumor progression [13] There is a complex interplay between B cells, TLS and CD4^+^ T cells, which may differentiate into several subsets of helper T cells (Th), including Th17 cells, which directly contribute to TLS formation, while also enhancing the cytotoxic effect of CD8^+^ cells in the presence of ICIs [14]. The prognostic and predictive value of B cells and TLS is increasingly recognized, but their roles in bladder cancer remain underexplored compared to T cells. This review aims to explore the emerging role of B cells and TLS in urothelial carcinoma.

## Biology of B cells in Cancer

B cells are lymphocytes of the adaptive immune system defined by expression of CD19, CD20, and surface immunoglobulin receptors and are a component of the TME in nearly every cancer. Major subsets exist in the context of cancer including naïve B cells, memory B cells, germinal center (GC) B cells, plasma cells, and B_regs_. The immune response involves B cells at various steps. Unprocessed antigens bind to B cell receptors (BCR) which activate B cell proliferation and differentiation into plasma cells. The antigens are internalized and degraded, ultimately being exposed to MHC class II molecules which initiate activation of CD4^+^ T cells. As such, B cells can be efficient antigen-presenting cells (APCs) [15]. A single-cell analysis in MIBC identified additional subtypes such as interferon-stimulated B cells and transitional B cells. These subsets, in addition to GC B cells are often spatially organized intratumorally within TLS, where GC B cells undergo clonal expansion, somatic hypermutation, and class switching, ultimately differentiating into plasma cells that secrete tumor-specific antibodies [16].

**Fig. 1:**
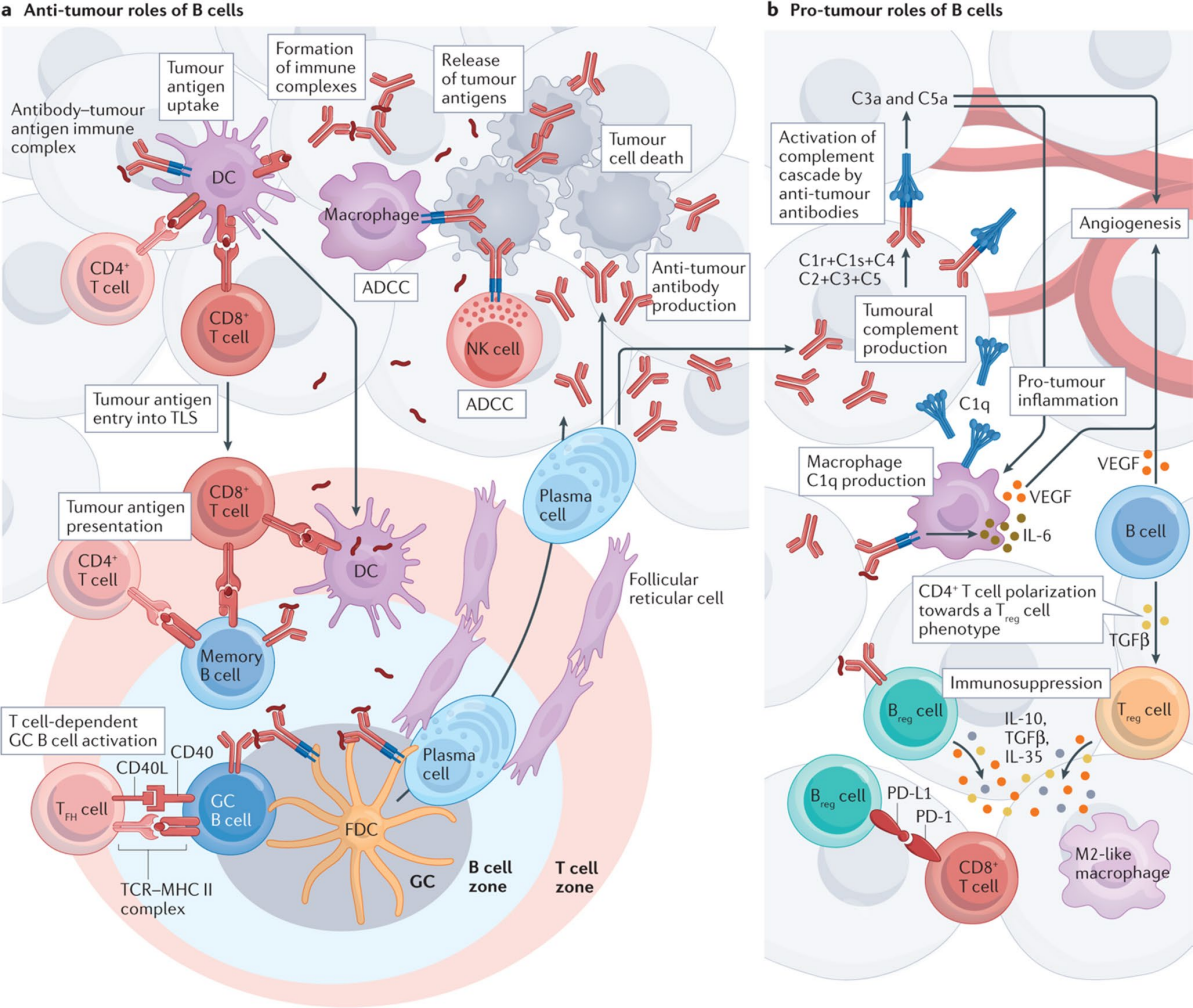
**a** The anti-tumour roles of B cells and tertiary lymphoid structures (TLS). In the T cell zone of TLS, mature dendritic cells (DCs) and potentially also B cells can present antigenic peptides to effector T cells, activating them to mount a response against tumour cells presenting the same antigens. Within the germinal centre (GC) in the B cell zone of TLS, follicular DCs (FDCs) present antigens in the form of immune complexes to B cells, which become activated and can subsequently differentiate into plasma cells. T follicular helper (T_FH_) cells also contribute to B cell activation in TLS. Plasma cells produced within GCs are propagated in the tumour bed and can produce anti-tumour IgG and IgA antibodies. In the tumour bed, natural killer (NK) cell and macrophage Fc receptors can bind to the constant regions of anti-tumour antibodies and induce antibody-dependent cellular cytotoxicity (ADCC) of tumour cells. The dying tumour cells release tumour antigens that can be taken up by DCs in the tumour microenvironment, which migrate to the TLS and present the antigens to lymphocytes. **b** Pro-tumour roles of B cells. Regulatory T (T_reg_) cells and B cells in the tumour microenvironment produce cytokines, such as IL-10, IL-35 and TGFβ, which impair the effector functions of T cells and skew macrophages towards an immunosuppressive phenotype, ultimately resulting in an inefficient anti-tumour immune response. Macrophages produce complement component 1q (C1q) that can bind to the Fc portion of anti-tumour antibodies and, in the presence of complement components C1r, C1s, C4, C2, C3 and C5 produced by tumour cells, activate the classical complement cascade, resulting in the generation of the anaphylatoxins C3a and C5a, which in turn trigger tumour-promoting inflammation and angiogenesis. Intratumoural B cells might also produce VEGF, which promotes angiogenesis. MHC II, MHC class II. Reprinted with permission. Originally published [18]

B cells appear to have dual roles in cancer: anti- and pro-tumor. Anti-tumor activity is primarily attributed to GC B cells, plasma cells, and interferon-stimulated B cells. Anti-tumor activity is typically defined by an “activated B cell phenotype” with intratumoral B cells [17]. A complex relationship between anti-tumor B cells stimulating CD4^+^ T-cells and cytotoxic CD8^+^ T-cells causes increased secretion of cytokines such as CXC-Chemokine ligand 13 (CXCL13) which attracts more B-cells to ultimately form a mature TLS as can be seen in Fig. 1a [18]. CXCL13, produced by mesenchymal organizer cells, follicular dendritic cells, and T follicular helper cells, signals by binding to its receptor, CXCR5, to drive lymphoid neogenesis and immune organization [19]. TLS derived plasma cells secrete immunoglobulins that enhance phagocytosis and support cytotoxic T cell responses, ultimately leading to a robust anti-tumor TME [16, 20].

Pro-tumor activity is mainly associated with B cells spatially located outside of the tumor, representing an “exhausted B-cell phenotype” [17]. B_regs_ are likely key contributors to tumor immune evasion, as seen in Fig. 1b. However, there is a lack of a specific marker to identify this cell type. B_regs_ are typically defined based on their function, secreting inflammatory cytokines such as IL-1B, IL-6, IL-21, or IL-35 [18]. Specifically, IL-10 + B_regs_ and TFG-β production by B_regs_ appear to be associated with and drive T cell differentiation to a regulatory T cell (T_reg_) phenotype [21]. B_regs_ are enriched in high-grade bladder tumors and contribute to immune evasion and disease progression. Transitional B cells and certain memory B cell subsets also display immunosuppressive phenotypes, with increased B_reg_ frequency correlating with more aggressive disease [22, 23].

## Tertiary Lymphoid Structures (TLS) in Cancer

TLS are ectopic, organized aggregates of immune cells that arise in non-lymphoid tissues under chronic inflammatory conditions, including cancer. Structurally, TLS resemble secondary lymphoid organs, featuring central B cell follicles (CD20+), surrounding T cell zones (CD3+), follicular dendritic cells (FDCs) (CD21+), and a network of high endothelial venules (HEV) and macrophages when fully mature. TLS development progresses through stages: initial loose aggregates of lymphocytes, termed ‘early TLS’, formation of distinct B and T cell zones, and maturation into structures with germinal centers capable of supporting local antibody production and affinity maturation [24, 25].

TLS formation is complex, orchestrated by chemokine-driven recruitment of lymphocytes and dendritic cells to the tumor microenvironment. Local production of the chemokine CXCL13 and the cytokine interleukin-7 (IL-7) recruits lymphoid, T helper 17, B, cells, and/or M1-polarized macrophages which interact with local stromal cells to form HEVs. Notable chemokines, such as CXCL12, CXCL13, CCL19, and CCL21, recruit lymphocytes which are delivered to the TME through HEVs and govern their organization into early TLS. As TLS mature, key cellular components include B cells (CD19+, CD20+), GC B cells, FDCs (CD21+, CD35+, CD23+, plasma cells (CD138+, CD269+), T cells (CD3+, CD8+, or CD4+), dendritic cells (DCs) (DC-LAMP, CD83+, CD86+), neutrophils (CD66b+), macrophages (CD68+), and HEVs (PNAd+, MECA79+) [26]. In a single-cell and spatial transcriptomics study, CXCL13 was identified in TLS formation in MIBC. In the proposed CXCL13-CXCR5 axis, CXCL13⁺ interacts with the CXCR5⁺ receptor on NR4A2 + B cells to form TLS. A higher proportion of CXCL13 + T cells was associated with better outcomes and a stronger anti-tumor response [16]. TLS are readily identified by Hematoxylin and Eosin (H&E) staining and may be further characterized by immunohistochemistry (IHC) [26].

Across multiple solid tumors, TLS density and maturation state have emerged as potential biomarkers of prognosis and IO response. Typically, the presence of mature TLS corresponds with favorable outcomes as has been evidenced in breast [27], colorectal [28], hepatocellular [29], lung [30], melanoma [31], ovarian [32], pancreatic [33], and stomach [34] cancers. Conversely, tumors lacking TLS or harboring immature aggregates tend to exhibit immune exclusion and therapeutic resistance. These findings position TLS as central features of the tumor immune landscape and potential therapeutic targets for reshaping antitumor immunity [24, 25]. There has been some mixed evidence in urologic malignancies. For example, TLS in MIBC generally correlates with improved response to IO while TLS in renal cell carcinoma (RCC) has a worse prognosis [35].

## B-Cells and TLS in MIBC

The immune microenvironment of MIBC has been studied using transcriptomics, single-cell sequencing, and spatial profiling, investigating a potential link between B cells, TLS, and clinical outcomes. Given the nature of muscle invasive disease, much of the data is derived from various clinical trials of systemic therapies. Additionally, there have been retrospective datasets that demonstrated B-cell infiltration and TLS density are associated with favorable prognosis in MIBC. A summary of relevant studies on B cells and TLS can be seen in Table 1. For example, Jiang et al. showed that high CD19⁺ tumor-infiltrating B cells correlated with improved overall survival and enhanced response to platinum-based chemotherapy, in part through their antigen-presenting role in priming CD4⁺ T cells [40].

**Table 1:** Summary of NMIBC and MIBC studies evaluating B cells and TLS

Author	Study Type	*N*	Main Findings
NMIBC			
Yilmaz and Sagir, 2024 [36]	Retrospective	135	TLS are frequently found in high-grade tumors and are associated with stage progression, but also improved response and favorable RFS to intravesical therapy
Ma et al, 2024 [37]	Retrospective	313 (NMIBC cohort)	TLS is associated with better DFS. However, multivariate analysis did not support this association
Balcik OY, Yilmaz F, 2025 [38]	Retrospective	96	FOXP3⁺/TLS presence was prognostic and predictive for patients undergoing treatment with BCG
Yolmo P et al, 2024 [39]	Pre-clinical	n/a	Tumor-adjacent TLS and immunosuppressive atypical B-cells were associated with poor response to BCG
MIBC			
Ma et al., 2024 [37]	Retrospective	267 (MIBC cohort)	TLS is associated with better DFS. However, multivariate analysis did not support this association
Jiang Q et al., 2019 [40]	Retrospective	246	Tumor infiltrating B cells served as an independent prognostic factor predicting for post-surgery survival and platinum-based chemotherapy benefit
Lin J et al., 2025 [13]	Retrospective	13	Elevated levels of B cells and TLS presence predicted improved OS in MIBC. A TLS gene signature may be feasible in predicting outcomes
Yuan H et al., 2024 [16]	Retrospective single-cell analysis	12	CXCL13 + T cells recruit CXCR5 + B cells which were critical in TLS formation. TLS presence correlated with favorable prognosis
Wang et al., 2024 [11]	Retrospective	26	TLS high patients have improved prognosis to PD-L1 therapy compared to TLS low patients
Pagliarulo F et al., 2022 [41]	Retrospective, IMvigor210/TCGA cohorts	692	TLS density and tumor mutational burden are independent prognostic biomarkers. High TLS density was associated with activated lymphocytes including intratumor B-cell and CD8 + T-cells
Aragaki AK et al., 2022 [42]	Retrospective, IMvigor210/TCGA cohorts	754	High levels of B cell and CD8 + gene signatures were associated with the longest OS and had improved clinical outcomes when treated with ICI, though these results were not consistent in women.
Groeneveld CS et al., 2016 [43]	Retrospective, IMvigor210/TCGA cohorts	348	CXCL13 correlates with the presence of TLS and was associated with improved survival in patients treated with ICI. CXCL13 may be a surrogate biomarker for TLS and ICI response

The predictive relevance of B cells and TLS have also been highlighted in ICI treated cohorts, as has been seen in the correlative science analysis of the IMvigor210 cohort. Gene expression analyses demonstrate that high TLS density and mature TLS gene signatures correlate with increased infiltration of activated B cells, CD8 + T cells, and T follicular helper cells, as well as upregulation of immune-related chemokines such as CXCL13. There was additional correlation with overall survival (OS) and higher rates of objective response to PD-1/PD-L1 blockade in MIBC [41].

Data from the NABUCCO trial, a pre-operative combination immunotherapy ipilimumab (anti-PD1) + nivolumab (anti-CTLA4) in patients with stage III urothelial cancer, has revealed additional nuance in B cell/TLS physiology. Co-expression of B-cell and CD8⁺ T-cell signatures (“B8T high/high”) identified patients with the most favorable outcomes, with sex-specific differences observed between men and women. Men receiving ICIs with B8T high/high tumors were associated with the longest survival, whereas in women the same B8T high/high profile did not confer a clear survival advantage. However, in ICI-naïve patients with MIBC, women with B8T high/high tumors had better OS. Although these findings require further validation, they suggest that the interaction between B-cell–rich immune niches and therapy can differ by sex [42].

TLS gene signatures and CXCL13 expression may serve as independent biomarkers for immunotherapy response and prognosis, outperforming other immune signatures and tumor mutational burden (TMB) in predictive value [43]. However, the combination of TLS metrics with traditional biomarkers may also improve prognostication. One study found that integrating TLS density with TMB into a joint TLS-TMB score stratified MIBC patient outcomes better than either alone. Interestingly, the composition of TLS (e.g. proportions of B cells, T cells, follicular dendritic cells) can vary, but differences in TLS cell makeup appeared to be dictated more by the surrounding TME context than by the maturation stage of the TLS itself [41]. Further, the physical relationship between immune cells within the TME may also impact clinical outcomes. Spatial transcriptomics demonstrated that proximity of TLS to CD8⁺ T-cell niches predicted response to combination PD-1/CTLA-4 blockade in urothelial carcinoma. Collectively, these studies position B cells and TLS as robust prognostic and predictive biomarkers for immunotherapy in MIBC [44].

In summary, the presence and maturity of B cell-rich TLS in MIBC are associated with favorable clinical outcomes and serve as prognostic and predictive biomarkers for immunotherapy response. Nonetheless, limitations include heterogeneity in TLS definition, lack of standardized quantification methods, and the need to distinguish effector from regulatory B-cell states. Addressing these gaps will be essential to translating TLS metrics into clinical practice.

## B Cells and TLS in NMIBC

Though most studies focus on MIBC, there is emerging evidence that B cells and TLS play an active role in NMIBC and may shape response to intravesical BCG therapy. A summary of relevant studies on B cells and TLS can be seen in Table 1. TLS can be readily identified in TURBT specimens and are more often found in high grade (HG) NMIBC tissue compared to low-grade (LG) NMIBC tissue [36, 45]. TLS also appear to be more common in HG invasive disease (*≥* T1 tumors) compared with HG non-invasive disease (Ta) [36]. From a biologic perspective, HG NMIBC will often invade if left untreated though it is worth noting that this disease state encompasses a wide range of phenotypes which limits the generalizability of the limited studies conducted thus far. As such, TLS presence in HG NMIBC is associated with more aggressive phenotypes if left untreated as this likely represents early invasive disease rather than a biomarker of poor response to therapy, as high TLS density in HG NMIBC also correlates with fewer recurrences after BCG therapy in several retrospective cohorts [36–38]. This apparent paradox can be reconciled by viewing TLS as a marker of an inflamed, antigen-rich microenvironment rather than of treatment resistance. IHC evaluating forkhead box P3 (FOXP3) (an anti-tumoral T cell marker) and T-cell immunoglobulin and mucin domain 3 (TIM-3) in conjunction with TLS presence found that elevation of these markers are associated with longer RFS in patients with NMIBC receiving BCG [38]. As a whole, retrospective data suggests that TLS presence is prognostic for more aggressive NMIBC with improved outcomes when treated with intravesical BCG.

However, the data is somewhat mixed. A carcinogen induced bladder cancer model in older mice has implicated atypical B cells (ABCs) in BCG resistance. Defined by CD19⁺CD21^lowCD11c⁺ phenotype and high self-reactivity, ABCs were shown to accumulate in the bladders of aging mice following intravesical BCG, particularly in female mice, which produced immunosuppressive cytokines and contributed to TLS with an exhausted phenotype. B-cell depletion in these models reduced TLS formation, enhanced CD8⁺ T-cell activity, and improved tumor control. Analysis of human NMIBC specimens suggested that TLS from BCG non-responders exhibited increased ABC-associated gene expression and immune exhaustion markers, linking this subset to poor outcomes [39]. However, these findings remain to be validated in larger human cohorts. Most clinical studies emphasize T-cell and myeloid dynamics, checkpoint expression, and overall immune contexture rather than B-cell phenotypes as determinants of outcome. Thus, while B cells and TLS are integral to the NMIBC immune landscape, the specific contribution of atypical B cells to BCG response remains uncertain and should be interpreted cautiously.

Additional research into the mechanistic interplay between B cells/TLS and local response to intravesical BCG are necessary to determine their precise role as a potential biomarker and therapeutic target.

## Future Directions

The emerging importance of B cells and TLS in bladder cancer raises opportunities for both biomarker development and therapeutic optimization. Some significant challenges remain in utilizing B cells/TLS as a reliable biomarker. Distinguishing protective vs. suppressive B-cell states remains a major challenge, particularly as B_regs_ do not have a reliable means of detection. Additionally, definitions of what constitutes a TLS and methods of quantification are inconsistent. Similar investigations in breast cancer have showed that simple H&E histology can underestimate TLS or confuse dense infiltrates for true structures with an inter-observer agreement coefficient of only R^2^ = 0.21 (95% CI 0.08–0.34) when H&E alone is use. This increased to R^2^ = 0.76 (95% CI 0.64–0.87) when IHC is used [46]. Adopting uniform criteria (through immunohistochemistry, gene signatures, or digital pathology) will be essential for reliably integrating TLS metrics into pathology reports and clinical decision-making. In gastric cancers, a machine learning model was developed and validated identifying TLS in > 95% of cases utilizing only H&E staining [47]. This has not yet been studied for validation in other malignancies.

Yet, given the improved response to IO in patients harboring mature TLS in multiple cancers, there has been interest is TLS induction as a therapeutic strategy. In a mouse ovarian cancer model, systemic delivery of CXCL13 successfully induced TLS formation and significantly improved survival by recruiting CD8⁺ T cells into the tumor [48]. In pancreatic cancer, co-injecting CXCL13 and CCL21 into the tumors of a murine model drove TLS neogenesis and markedly enhanced the anti-tumor effects of chemotherapy [49]. Current work by our own group is underway using a murine model of NMIBC and intravesical CXCL13 to induce TLS formation. These findings suggest that modulating chemokine pathways to promote TLS formation could help convert “cold” tumors into “hot” immune phenotypes, thereby improving responses to immunotherapy. Inducing TLS may be most useful in tumors lacking spontaneous lymphoid structures, whereas in “hot” tumors the focus could be on sustaining TLS function. While TLS and B-cells are typically secondary outcomes, intravesical dendritic targeting of Fc-enhanced CD40 agonists can recruit B cells and induce TLS [50, 51]. In MIBC, a phase Ib trial utilized an intravesical oncolytic immunotherapy cretostimogene grenadenorepvec and systemic nivolumab and found that the addition of the intravesical oncolytic immunotherapy seemed to enhance the formation, enlargement, and maturation of TLS [47].

## Conclusion

B cells and tertiary lymphoid structures are heterogenous and dynamic components of the bladder TME that can shape the prognosis and therapeutic response in both NMIBC and MIBC. B cells may play an anti-or pro-tumor role depending on their phenotype. Anti-tumor B cells often form TLS, and the presence and maturity of TLS consistently associate with improved outcomes in MIBC, though have mixed results in NMIBC. Moving forward, standardization of TLS assessment, integration of B-cell/TLS biomarkers into patient stratification, and exploration of TLS-inducing strategies have the potential to move from exploratory findings to clinically actionable tools that guide personalized therapy in bladder cancer.

## Key References

- Yuan H, Mao X, Yan Y, Huang R, Zhang Q, Zeng Y, et al. Single-cell sequencing reveals the heterogeneity of B cells and tertiary lymphoid structures in muscle-invasive bladder cancer. J Transl Med. 2024;22[52]:48. 10.1186/s12967-024-04860-1.
  - ○ Identified distinct B cell subtypes and identified TLS as a prognostic biomarker in MIBC.
- Jiang Q, Fu Q, Chang Y, Liu Z, Zhang J, Xu L, et al. CD19(+) tumor-infiltrating B-cells prime CD4(+) T-cell immunity and predict platinum-based chemotherapy efficacy in muscle-invasive bladder cancer. Cancer Immunol Immunother. 2019;68[52]:45-56. 10.1007/s00262-018-2250-9.
  - ○ B-cells within bladder tumors are assocaited with improved response to adjuvant chemotherapy.
- Wang X, Juncker-Jensen A, Huang G, Nagy ML, Lu X, Cheng L, et al. Spatial relationship of tertiary lymphoid structures and tumor-associated neutrophils in bladder cancer and prognostic potential for anti-PD-L1 immunotherapy. Cancer Commun (Lond). 2024;44(4):499-503. 10.1002/cac2.12491.
  - ○ Mature TLS are associated with improved response to IO.
- Groeneveld CS, Fontugne J, Cabel L, Bernard-Pierrot I, Radvanyi F, Allory Y, et al. Tertiary lymphoid structures marker CXCL13 is associated with better survival for patients with advanced-stage bladder cancer treated with immunotherapy. Eur J Cancer. 2021;148:181-9. 10.1016/j.ejca.2021.01.036.
  - ○ CXCL13 can serve as a biomarker for presence of TLS and is associated with improved survival in patients with MIBC treated with IO.

## Data Availability

No datasets were generated or analysed during the current study.
